# Mitochondria and Cancer: Past, Present, and Future

**DOI:** 10.1155/2013/612369

**Published:** 2013-01-28

**Authors:** M. L. Verschoor, R. Ungard, A. Harbottle, J. P. Jakupciak, R. L. Parr, G. Singh

**Affiliations:** ^1^Juravinski Cancer Centre, 699 Concession Street, Hamilton, ON, Canada L8V 5C2; ^2^Department of Pathology and Molecular Medicine, McMaster University, 1280 Main Street W, Hamilton, ON, Canada L8N 3Z5; ^3^Mitomics Inc., Bioincubator Suite, The Medical School, New Castle University, Framlington Place, Newcastle Upon Tyne NE2 4HH, UK; ^4^Cipher Systems, Annapolis, MD 21401, USA; ^5^Mitomics Inc., 290 Munro Street, Suite 1000, Thunder Bay, ON, Canada P7A 7T1

## Abstract

The area of mitochondrial genomics has undergone unprecedented growth over the past several years. With the advent of the age of omics, investigations have reached beyond the nucleus to encompass the close biological communication and finely coordinated interactions between mitochondria and their nuclear cell mate. Application of this holistic approach, to all metabolic interactions within the cell, is providing a more complete understanding of the molecular transformation of the cell from normal to malignant behavior, before histopathological indications are evident. In this review the surging momentum in mitochondrial science, as it relates to cancer, is described in three progressive perspectives: (1) Past: the historical contributions to current directions of research; (2) Present: Contemporary findings, results and approaches to mitochondria and cancer, including the role of next generation sequencing and proteomics; (3) Future: Based on the present body of knowledge, the potential assets and benefits of mitochondrial research are projected into the near future.

## 1. Past

As far back as 1850 scientists identified the existence of structures within cells that today we call mitochondria [1]. However, it was not until 1898 that the these structures were given the term mitochondria by Carl Benda [2]. Cytologists worked hard to identify the function of mitochondria and in 1912 the first reference to a possible link between mitochondria and respiration was made by Kingsbury [2]. This link was made exclusively from morphological observations. What followed was 30–40 years of intense biochemical analyses before the characterization of mitochondria as the “Powerhouse of the Cell” by Siekevitz in 1957 [3]. Leading up to this there were a number of key events. In 1909 Correns and Baur independently identified the first cases of extracellular inheritance. The source and site of this was unknown at the time, though mitochondria were the prime suspect. Ephrussi's laboratory had been working with yeast and their key publication in 1949 [4] used genetic analysis to show that respiration-deficient baker's yeast harboured mutations found in the cytoplasm, and not the nucleus. Soon thereafter Slonimski and Ephrussi investigated this area further and showed the deficiency was due to mitochondrial dysfunction.

 On the back of these exciting findings, one of the most important discoveries in mitochondrial research was made in 1963, when the identification of the existence of mitochondrial DNA (mtDNA) was made by M. M. Nass and S. Nass [5]. Using electron microscopy they showed conclusively that chick embryo mitochondria contained DNA. The importance of this discovery cannot be overstated; as it renewed interest in the evolutionary origin of mitochondria. These findings were confirmed biochemically in 1964 when Schatz and Klima [6] showed that baker's yeast mitochondria also contained DNA. This of course led to more questions, specifically does the mtgenome interact with the nuclear genome, and if so how does this occur? 

 The mtDNA sequence was first published as being 16,569 base pairs long in 1981 by Anderson et al. [7], the sequence was later revised by Andrews et al. in 1999 [8]. Following the publication of the mtDNA sequence there was a focus on mitochondrial genomics that has been sustained till today. This work initially focused on myopathies and neuropathies by Wallace et al. [9, 10]. These so called “mitochondrial diseases” were due to both mitochondrial and nuclear mutations, where a symbiotic relationship exists between the mitochondrial and nuclear genomes. Understanding the complexity of these interactions is the key to detecting dysfunction within the cell. 

 Mitochondria control various metabolic functions and synthesizes 95% of cellular metabolic energy, while 1,200 nuclear genes drive and participate in mitochondrial function. There are 37 genes coded for by the mtgenome, 24 of which are dedicated to processing 13 genes within the mtgenome (mtgenome) itself which produce the subunits essential to electron transport. These 13 key genes work in conjunction with 93 nuclear proteins. In cancer cells certain mutations in the mtgenome can alter the biochemical behaviour of mitochondrial/nuclear protein complexes, thereby increasing pools of reactive oxygen species (ROS) which in turn enable tumour growth and may provide proliferative advantage to the cell [11]. Thus despite its small size relative to the nuclear genome, somatic mutations that occur in the mtgenome are able to contribute directly to the process of tumourigenesis. 

 There is now a significant body of literature describing the interactions between mitochondria and the nucleus. It appears that the somatic mutations which can alter these interactions occur early in the disease process, appearing in histologically normal tissues [12]. This leaves us with a key question with regard to mitochondrial mutations, are they causative factors of disease or a simple record of the development of disease? In 1924 Otto Heinrich Warburg postulated that cancer and tumour growth are in part caused by a change in the way the cells generate their energy. In normal healthy cells, ATP is generated mainly by oxidative breakdown of pyruvate following non-oxidative breakdown of glucose during the process of glycolysis. In contrast, malignant cell metabolism stalls after glycolysis a phenomenon that Warburg reported as the fundamental difference between normal and cancerous cells. These observed differences in the ratio of glycolysis to respiration have become known as the Warburg effect [13]. It has since become clear that these metabolic differences adapt cancer cells to the hypoxic conditions inside solid tumours. Thus it may be theorized that rather than causing cancer, perhaps these changes are characteristics produced by key cancer-causing mutations in certain genes involved in the aforementioned symbiotic relationship between nucleus and mitochondria. These findings have led to a new theory known as the Warburg theory of cancer, which suggests that the main driver of tumorigenesis is an insufficient cellular respiration caused by insult to mitochondria [14]. 

 Clearly mitochondria are key to the function of both normal and malignant cells, and there has been much speculation about the origins of mitochondria, with perhaps the most prevalent theory being that of an endosymbiotic origin. Mitochondria have many features in common with prokaryotes, and this theory is generally accepted today. At its very basic level, the endosymbiotic theory hypothesises that mitochondria, chloroplasts and perhaps other organelles of eukaryotic cells, originated as independent organisms which were taken inside what became eukaryotic cells as endosymbionts. It has been suggested that mitochondria as we know them today developed from proteobacteria, specifically the SAR11 lineage [15]. Although the endosymbiotic theory has been around for over one hundred years, it remains a controversial and developing hypothesis with new evidence for and against the theory still appearing today. Although beyond the scope of this publication, this controversy certainly highlights mitochondria as a hot topic for research in many diverse fields of study.

## 2. Present

Mitochondria play a central role in the regulation of cellular function, metabolism, and cell death in cancer cells. Several important functional changes to cancer cell mitochondrial have been observed that implicate the organelle in tumour formation including increased production of reactive oxygen species (ROS), decreased oxidative phosphorylation, and a corresponding increase in glycolysis [16, 17]. However, the specific role of mitochondria in tumourigenesis remains unclear as these changes could represent either key mechanisms in tumour initiation, promotion, or simply secondary effects of tumourigenesis. In 2011, deregulated cellular energetics was considered as an additional emerging hallmark of cancer [18]. Cancer cell signaling that is regulated by kinases and phosphatases are guided by cellular redox status and may be a key in malignant transformation. This section briefly examines the role that mitochondria play in the cancer cell phenotype by relating the physiological process of the organelle to genomic and proteomic studies.

### 2.1. Role of Mitochondria in Oncogenesis

Recent studies of mitochondrial involvement in cancer have uncovered a plethora of differences in the structure and function of these organelles upon comparing metastatic mitochondria to those belonging to nontransformed cells. Notably, modern research has largely upheld the metabolic observations of Warburg and his successors, while refining and greatly expanding the breadth of mechanistic knowledge of mitochondrial state and function in tumour development. The comprehensive mechanisms of the Warburg effect have not yet been isolated; however, multiple intertwining causative and responsive mechanisms have recently been characterized. This understanding of the indicative features of cancer cell metabolism has also directly been applied to current clinical care through the increasingly widespread adoption of positron emission tomography (PET) imaging using glucose analogues to identify cancerous lesions that are characterized by high glucose uptake [19].

Study of the mechanisms of the Warburg effect has revealed that the characteristic metabolic shift towards aerobic glycolysis and increased glucose uptake imparts several functional advantages to the cancer cell. These advantages permit rapid growth and survival in conditions that would be potentially lethal to noncancerous cells. Perhaps the most significant shift in the understanding of the Warburg effect in recent years has been the abandonment of the view that aerobic glycolysis is a metabolic defect of cancer cells, in favor of the theory that cancer cell metabolism is maintained through regulatory control, and better fits the metabolic profile of rapidly dividing cells [20]. The most recognized of these adaptations is the utilization of abundant glycolytic pathway intermediates in multiple anabolic reactions critical to the survival and growth of rapidly dividing cells. The demand for glucose-derived carbon skeletons for macromolecule synthesis of molecules such as glycogen, phospholipids, triglycerides, and malate, exceeds the demand for efficient ATP production [21]. Glucose is also alternatively metabolized in cancer cells through an enhanced pentose phosphate pathway that results in the synthesis of nucleotides and antioxidant nicotinamide adenine dinucleotide phosphate (NADPH) [20].

The cancer cell's lack of reliance on oxidative phosphorylation for ATP generation also permits cellular survival in conditions of inconsistent oxygen supply, an environment that is typical for rapidly expanding tumours which can, at times, experience inadequate angiogenesis [22]. Local acidification of the tumour microenvironment is also induced through the glycolytic generation of excess bicarbonic and lactic acids. The resultant pH change is recognized to favour tumour growth and invasion through the activation of cancer cell-derived cathepsins and metalloproteinases [23], and the inhibition of the subsequent host immune responses [24]. Additionally, the ensuing excessive production of lactate can be converted to pyruvate in cancer-associated stromal cells to fuel oxidative phosphorylation within these cells [25]. 

It is also well established that the enhanced production of mitochondrial ROS, most notably superoxide, hydroxyl radicals, and hydrogen peroxide, is a prominent byproduct of cancer cell metabolism. Increased oxidative phosphorylation in pre-metastatic cells therefore increases the production of mitochondrial ROS, which may be an initiative factor in carcinogenesis [26]. The mechanisms of ROS production and their significant downstream effects have become important topics in current mitochondrial research in cancer. Excess ROS act not only as mutagens and initiators of oxidative stress, but are also significant inter- and intracellular signaling molecules, responsible for a host of nuclear and mitochondrial changes in gene expression, the details of which are reviewed by Verschoor et al. [27].

The well-established addiction to glutamine as an energy source of proliferating cancer cells is yet another key hallmark of cancer cell metabolism. In the cytoplasm, glutamine is converted to glutamate by glutaminase, and transported into the mitochondria where it is converted to the TCA cycle intermediate *α*-ketoglutarate, and also acts as a source of substrates for macromolecule synthesis [28]. Glutamate is also a key substrate of the glutathione-dependent antioxidant system that is the primary intracellular antioxidant mechanism, and that is critical to cellular protection from ROS. Recent studies have also shown that glutamine is an important component in several signaling pathways involved in cell growth including mTOR and ERK pathways, and glutamine uptake and degradation are controlled via c-myc regulation [29].

The increased mitochondrial ROS production in metastatic cells has also been associated with the corresponding upregulation of cellular antioxidant defense mechanisms [30]. In particular, the synthesis of GSH is significantly increased by the enhanced uptake of rate-limiting cystine through the action of the cystine/glutamate antiporter system *x*_*C*_^−^, which is frequently upregulated in cancer cells [31]. This increased antioxidant capacity has been implicated in cellular resistance to chemotherapy and radiation therapy that induce cancer cell death by initiating oxidative stress [32]. In addition, it was recently found that the abundant glutamate excreted as a byproduct of cystine uptake by system *x*_*C*_^−^ in cancer cells may also initiate several significant pathologies of metastatic tumours including excitotoxic cell death in tumours of the CNS, and disruption of bone cell signaling in metastatic tumours in the bone [33, 34].

### 2.2. Genomics of Mitochondrial DNA: Mutations and Polymorphisms

The circular mtgenome encodes 37 genes including several components of the electron transport chain (ETC), tRNAs, and rRNAs. Additionally, mtDNA contains a non-coding region comprised of two hypervariable regions within a displacement loop (D-loop), which is the location of the origin of replication and transcriptional promoters. Mutations in mtDNA are frequently observed in cancer, likely due to the lack of introns, lack of histone protection, and close proximity to damaging ROS. Each cell contains multiple copies of mitochondrial genes, giving rise to mitochondrial homoplasmy, where all the mitochondria of a cell have the same genomic composition, or heteroplasmy, where wild-type and mutant mtDNA coexist [35]. Thus is it possible for a mutation that confers a distinct advantage for cancer cells, such as accelerated growth or enhanced survival, to be clonally expanded to become a homoplasmic mutant and to predominate within a population of cancer cells. Alternatively, Coller and colleagues [36] used a mathematical model to show that random segregation of mtgenomes during rapid tumour development could result in a mutant homoplasmic population without the need for a selective advantage. Regardless of the existence of background homoplasmic mutations that confer no functional consequence, there are numerous mtDNA mutations that result in significant alterations in mitochondrial function that affect tumour development and progression. 

In certain cancer tissues mtDNA mutations were more readily detectable and abundant than mutated nuclear p53 DNA, suggesting that mtDNA mutations could serve as excellent cancer biomarkers, particularly for early detection [35]. The most commonly mutated or deleted region of mtDNA in cancer is within the D-loop at the D310 tract, which is a mononucleotide cytidine repeat at position 310 [37]. As the D-loop is involved in mitochondrial replication, mutations in this region could also affect mtDNA copy number, though this theory has yet to be proven empirically. In one study, colorectal cancer patients with D-loop mutations were found to have significantly lower overall survival rates and increased chemotherapeutic resistance compared to patients who's mtDNA did not harbour such mutations [38]. The high frequency of D-loop deletion or insertion somatic mutations in cancer render these mutations unlikely to confer any functional impairment to mitochondria, and so the uncertain functional consequences of these mutations should remain an important area for mitochondrial research in cancer.

The importance of mitochondrial polymorphisms in cancer development and risk is intimately related to evolutionary haplogroups, and has recently been a contentious area of research. Haplogroups are characterized by a specific mutation that occurs widely within individuals of a particular population, and are further divided into haplotypes generally based on restriction fragment length polymorphisms [39]. Among the main European haplotypes, the A12308G mutation in tRNA^Leu2^ common to haplotype U was associated with increased risk of both renal and prostate cancers [40]. The NADH-ubiquinone oxioreductase chain 3 (ND3) substitution mutation at G10398A has been associated with increased breast cancer risk in both African American and Indian women [41–45]. In European-American women the A10398G ND3 substitution conferred increased risk of breast cancer, as did the T16519C D-loop polymorphism [46]. A comprehensive study of pancreatic cancer risk revealed associations with the A331T substitution in mitochondrial ND2 [47]. Despite these promising findings, and because the majority of mtDNA polymorphisms are functionally inconsequential, associations with specific polymorphisms and cancer risk have been subject to heated debate. Several older studies involving association of specific polymorphisms with cancer risk have been heavily scrutinized due to erroneous experimental design, interpretation, and poor data quality [35]. However, due to the potential usefulness of somatic mtDNA mutational profiling as a diagnostic tool, the study of mitochondrial somatic mutations and associations with cancer should remain an important focus of cancer biomarker research pending proper study design, population stratification, and independent replication of results. Interestingly, one study reports that one well characterized pathological mtgenome alteration, A3243G, drives mtDNA depletion [48]. 

Mitochondrial depletion, a hallmark of cancer initiation and malignant development, is characterized by a wide range of mtDNA deletions [49, 50]. In prostate cancer, a cascade of both large and small-scale deletions reduce cellular mtDNA. This reduction is associated with androgen independence which facilitates disease progression [51]. Consistent with these findings, a 3.4kb mtgenome deletion is currently being used by many urologists to identify the presence and/or absence of prostate cancer in patients with an initial benign biopsy [52, 53]. Contrary to a negative outcome these patients remain highly suspicious for disease by other clinical parameters. 

### 2.3. Mithondrial Genome Sequencing

Homoplasmic and heteroplasmic mutations have been reported in the mtgenomes of patient tumors [54], and improved patient outcomes have been demonstrated using mtDNA mutation identification for early detection of solid tumours [55]. Clinically, the detection of mtDNA mutations could be reliably used to compare differences in healthy and cancerous tissues, used to monitor mutations in high-risk, asymptomatic patients, or to monitor cancer patients for recurrence of the disease. Although mtDNA mutations have been reported in a wide variety of human cancers extending early detection to cancer prevention has proved problematic with regards to linking homoplasmic or heterplasmic variations with the etiology of cancer. Thus the characterization of populations of mtDNA variation would facilitate broad acceptance of mtDNA analysis.

 Initial studies on whole mtgenome analysis established protocols for directly sequencing entire mtgenomes to detect sequence changes. For example, age-matched individuals with lung cancer had strikingly different mtgenome signatures, suggesting that these variants could be cancer-associated changes [56]. To evaluate progression of mtDNA mutation load associated with tumor stage progression, mtDNA mutation type and location across the entire mtgenome were evaluated between individuals with different stages and different types of cancer. Sequence variants were identified in stage I to stage IV tumor samples, and these mutations were distributed across the entire mtgenome with no indication of a hotspot or specific site of mutation associated with specific cancer types or stages. Analysis across larger genome regions indicated a significant clustering of mtDNA mutations in the ND gene complex, while 10% of mitochondrial mutations were found in the D-loop region. 

**Figure 1:**
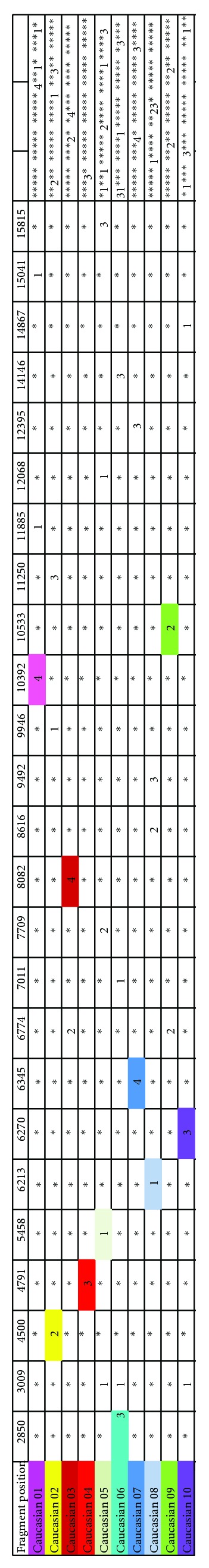
Numbered position of location of mutations in the mtgenome is listed across the top. Transformation of sequence information to a number enables a bar-code description of the samples. The HV regions do contain mutations, but they are identical and hence of no informative value and are not shown. On the contrary, mutations across the entire mtgenome demonstrates that whole genome analysis has clear utility.

 The importance of whole genome analysis can be recognized in analogous measurements of entire mtgenomes. In human forensics, sequencing entire mtgenome is more effective because polymorphisms in mtgenomes can be useful for resolving individuals who have the identical hypervariable (HV) HV1 and HV2 control region sequences [57]. Using the whole genome as a potential source of mutations improves the discrimination power of forensic assays [58], and by extension cancer diagnostics, prognostics and tumor profiling. Figure 1 illustrates the advantage of whole genome analysis. All ten samples have identical D-loop mutation patterns and types, thus these samples are not distinguishable with only partial mtgenome analysis. As whole mtgenome sequencing is ubiquitous, easy to perform, and high-throughput for even small genomes, whole mtgenome sequencing should be the standard. 

### 2.4. Considerations for Mitochondrial DNA Analysis

**Figure 2:**
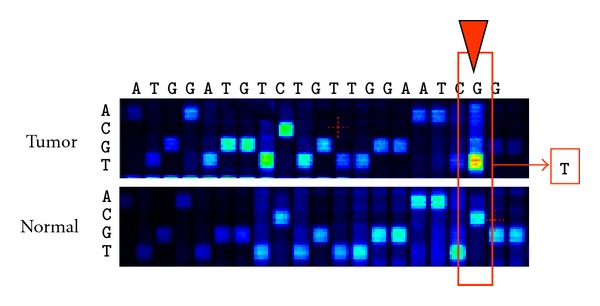
Samples were analyzed by whole genome sequence comparison of (1) tumour tissue and (2) matched patient blood. The sequence of a small region of the mtgenome is indicated across the top of the figure. The red box indicates position and type of mutation observed in the tumour specimen.

Proper analysis of mtgenome mutations is required for accurate correlation of homoplasmic mutations with tumor tissue and stage (Figure 2). Mutations are detected by comparing DNA sequence of tumor tissue to that of normal tissue or blood from the same individual [59]. It is important to use blood as control tissue measurements because it is necessary to subtract out mtDNA variation that arises from accumulation of damage over the lifespan, for example, due to aging. Direct analysis of haplogroups associated with cancer does not establish a correlation with variation of mtgenomes metastasis [60]. Comparison of control tissue to tumor tissue must be conducted with such samples collected from the same individual. Accurate detection of mtDNA mutations must account for other sources of errors, for example nuclear mitochondrial pseudogenes (numts) are sources of contamination during PCR amplification. This warrants careful experimental design and cautious interpretation of heteroplasmic results. Hence, mtgenome disease-associated biomarkers must be authenticated to preclude false-positive detection of paralogous nuclear pseudogenes [61].

### 2.5. Cancer Field Effect

Another important reason to characterize entire mtgenomes is because of occurrence of mtDNA mutations that could be part of a cancer field effect within tissue. These mutations could be biomarkers for progressive mutation patterns in lesions. However, correlation between single mutation sites and specific gradings remains loosely associated. Instead of attempting to define hotspots for mutations, the gradual accumulation of mutation(s) distributed across entire genomes could be considered as an “individualized” marker of progression. Studies have reported no correlation of tumor-associated mtDNA mutations with respect to patient age. The mutation load or population are uniquely attributed to each starting point, and thereafter constrained by risk and rate based on initial populations. While mutation load is individualized, DNA damage may not be compartmentalized to one site. Although low sensitivity assays and limited sampling have plagued the majority of mtgenome comparative studies, not every tumor possessed sequence variants, while some samples contained a number of variants [58, 62]. 

 Analysis for identical damage in different tissues (cancer versus control) could be more apparent with analysis of populations of mtgenomes that indicate tissue predisposed to cancer. Absence of a 1 : 1 correlation between the mutation patterns of tumor progression is likely a result of tissue sampling. Hence, population analyses would facilitate characterization of specific locations and surrounding tissues. In summary, there are salient considerations for mtDNA mutation identification comparative studies between potential cancer tissue, different types and stages of tumors, as well as non-invasive collected samples and experimental controls. It is imperative to incorporate analysis of the entire mtgenome and accurate identification of heteroplasmic mitochondrial populations. Additionally, the sampling of bodily fluids and tissues surrounding cancerous tissue will facilitate defining the extent of the cancer field effect.

 The derivation of sequence changes in the mtgenome in cancer remains unclear. Should these changes prove to be clonal expansions of a heteroplasmy already present in the tissue rather than tumour-associated *in situ* mutations, early detection of cancer may thus rely on the ability to differentiate levels of heteroplasmy. In general, studies of mtDNA mutations in cancer indicate the presence of sequence variants spanning the entire mtgenome, and therefore full genome sequencing will provides the cancer diagnostic community with a useful biomarker discovery approach. Such characterization of populations would be useful to define PCR panels for inexpensive triage and screening of human populations. The sensitivity of mutation detection, rates as little as 2% contribution to the admixture of normal and tumor DNA, indicate heterogeneous biological samples such as bodily fluids, lavage specimens, fine-needle aspirates, or biopsies can potentially be analyzed for cancer-associated mitochondrial DNA mutations. The identified heteroplasmic and homoplasmic sequence variants from tumors and blood (control) and urine (for matched bladder cancer) and bronchoalveolar lavage (for matched lung cancer) were measured from the same individual. It is a reasonable assumption that heteroplasmies should comprise multiple subpopulations of mutated mtDNA molecules. Research that incorporated whole mtgenome analysis using sensitive methods describes sequence variant identification of both heteroplasmic and homoplasmic sequence variants in clinical samples distributed across the mtgenome. Hence, small cohort studies that use incomplete mtgenome sequencing or methods designed to scan discrete portions of the genome, miss important sequence variants associated with cancer or other diseases. It is now possible to screen populations to understand specific frequencies and distributions, and to compare sequences of entire mtgenomes in order to have a comprehensive characterization of sample material. Finally, on account mtDNA mutations are well validated, mitochondrial research is beginning to concentrate on understanding the link between mitochondrial function and pathological states. There are a few studies that have begun to address the association of mitochondrial function with change in homeostasis [63, 64], as well as mitochondrial redox state [65]. As technology improves to allow the accurate assessment of cellular interaction and hemodynamics, monitoring the effects of mitochondrial dysfunction in combination with using mtgenome mutation diagnostics on the pathophysiology of cancer cells will begin to support medical decision-making. 

### 2.6. Mitochondrial Proteomics

The majority of mitochondrial proteins are encoded by the nuclear genome and imported to the mitochondria to perform their specific functions. Thus the mitochondrial proteome is the result of complex crosstalk between both nuclear and mitochondrial programs, and is greatly influenced by pathological conditions including cancer. In the past decade, the mitochondrial proteome has been characterized from highly purified mitochondria resulting in a comprehensive list of over 1,000 mitochondrial proteins (as reviewed in [66]). Using the wealth of knowledge from such studies, numerous databases have been created such as MitoInteractome [67], MitoP2 [68], HMPDb, and MitoMiner [69]. The MitoInteractome database contains 6,549 protein sequences derived from mutltiple databases (SwissProt, MitoP, MitoProteome, HPRD, GO) from several different species creating a comprehensive protein-protein interaction network. Certainly one of the most extensive databases, MitoP2 contains data from a wide breadth of mitochondrial proteomic studies spanning from single protein studies to extensive proteome-wide mapping and expression studies. The HMPDb (Human Mitochondrial Protein Database) provides consolidated information on mitochondrial DNA sequences, polymorphisms, disease-related proteins, and 3-D mitochondrial protein structures. Collectively these databases serve as wonderful utilities for the discovery and characterization of novel mitochondrial biomarkers for diagnosis and molecular targets for drug treatments.

 Extensive protein expression differences have been found in mitochondrial glycolytic enzymes, heat-shock proteins, cytoskeleton proteins, and antioxidant enzymes through comparative proteomic analysis. In regards to metabolism, proteins of the glycolytic and pentose phosphate pathways tend to be induced, along with reductions in oxidative phosphorylation pathways [66, 70]. Recently, Chen et al. (2011) performed 2D-DIGE and MALDI-TOF mass spectrometry to compare the proteomic profile of purified mitochondria from normal breast cells (MCF10A), non-invasive breast cancer cells (MCF7), and invasive breast cancer cells (MDA-MB-231) [71]. The most differentially expressed mitochondrial proteins between normal and cancerous cells included cytochrome oxidase subunit 5B, malate dehydrogenase, and elongation factor Tu. Several proteomic studies have shown a significant correlation between high levels of heat-shock protein 70 (HSP-70) in a variety of cancers including gastric adenocarcinoma, hepatocarcinoma, and oesophageal cancer [66]. HSP-70 functions as a mediator of cell proliferation, cellular senescence, and cellular immortalization, and when concentrated in to cytoplasm sequesters p53 and activates Ras-Raf signaling which controls cell proliferation [72, 73]. 

Recent observations and interest in mitochondrial research has generated a lot of enthusiasm and hope that novel therapeutic agents will be identified that are effective for cancer therapy. In a general sense a dynamic and bidirectional exchange between the metabolic status of the cell generated by mitochondria and genetic profile of a cell will provide a better understanding of metabolites and unexplored signaling mechanisms. Hence a complete understanding of the mitochondrial proteome and its regulation by metabolites, including ROS, will provide a better understanding of the symbiotic relationship that has evolved in eukaryotic cells. Additionally, recent advances in high-throughput technologies, such as next-gen sequencing and Mitochip [74], have allowed for the rapid and accurate detection of mtDNA mutations, polymorphisms, or copy number variations in a variety of tissues and bodily fluids [55, 75–79].

## 3. Future

The mitochondrion, as the biochemical nexus of the cell, is a critical consideration in the genomics era of the new millennium. Although much of the current funding is aligned to continuing to further understand the functional details of the nuclear genome, the mitochondrion and its modest complement of DNA and protein is emerging as a crucial component of the biological networking of nuclear pathways. In addition to generating 95% of the chemical fuel firing cellular metabolism through carbohydrate breakdown, mitochondrial perform and mediate a number of events including ROS generation, retrograde calcium signaling and intrinsic apoptosis. Most of these pathways are fundamentally altered during malignant transformation. For example, the apoptotic pathway is severed and carbohydrate metabolism is preempted for anaerobic respiration. Mitochondria shroud a multitude of unidentified proteins, suggesting yet to be understood, deeper biological functions [80]. This important information is not yet fully detailed; however, it is rich with promise. It is likely that these discoveries will provide new approaches to cancer treatment, diagnosis and prognosis. For example, the accelerated mutation rate of the mtgenome offers early identification of malignant transformation by identification of a field effect in normal appearing tissue [81]. This molecular conditioning is well attested particularly in prostate cancer [52, 82, 83]. In addition, the mtgenome has a high copy number and an increased somatic mutation rate in comparison to its nuclear counterpart, providing multiple target copies with target markers. This threesome of characteristics (field effect, copy number, mutation rate) will enable monitoring of vulnerable epithelium in organs such as the lungs, colon, breasts, prostate, and ovaries. Resection of both tumors and the surrounding field may have important implications for recurrence [79]. Since malignant transformation is a 20 to 30 year process, in most cases a shift to a field effect could allow prediction of the development of intraepithelial neoplasia (IEN). Specific markers may indicate which IEN lesions and molecular renovations may progress towards a malignant phenotype. The American Association for Cancer Research (AACR) Task Force on the Treatment and Prevention of IEN published the following statement in 2002:

* “The AACR IEN Task Force recommends focusing on established precancers as the target for new agent development because of the close association between dysplasia and invasive cancer and because a convincing reduction in IEN burden provides patient benefit by reducing cancer and because a convincing reduction in IEN burden provides patient benefit by reducing cancer risk and/or by decreasing the need for invasive interventions [84].”*

In addition, the traits of mtDNA have successfully led to identification of mitochondrial mutations in low cellular biofluids such as nipple aspirate fluid [85]. Significant resolution between bladder cancer stages Ta, T1 and T2 was obtained using the SNP counts in whole mtgenome sequencing of urine cell pellets in 20 of 31 patients [86]. Notably, circulating cell-free mitochondrial DNA in peripheral blood has diagnostic utility for breast cancer, urological malignancies, and predicting prostate cancer recurrence. [87–89]. 

Due to its central role in cell physiology, specific alterations in the mtgenome may indicate the status of specific pathways or impact biological outcomes. For example, mutations in mitochondrial respiratory complexes may influence the induction of apoptosis [90] and promote metastatic behavior in both prostate and breast cancers [49]. These studies suggest that the sequence of bases in the mtgenome are finely ordered to the point that even some sequence specific haplogroups may be more susceptible to malignancies [50]. This concept should not be surprising since “natural selection mediated by climate has contributed to shape the current distribution of mtDNA” [91]. Hence mitochondria are dynamic, adaptable molecules able to mitigate biological compromise given metabolic parameters. Disease susceptibility may be tolerated due to imposed climatic constraints. 

The cellular ganglion of mitochondria, plethora of pathways and high volume molecular trafficking have been recognized as ideal chemotherapeutic targets [52]; however, this approach draws the proverbial “double-edged sword.” For instance, the adjuvant treatment of estrogen receptor positive breast cancer with tamoxifen requires intact and fully operational mitochondria [92]. Importantly, mitochondrial toxicity is a major implication in the failure of chemotherapeutic agents in the late stages of drug development [93]. Careful consideration of mitochondrial and compound interactions is imperative to both target mitochondria for therapeutic indications, while avoiding off-target effects of other therapeutic molecules. Disruption of key mitochondrial molecular transport molecules, such as SCaMC-1, or SLC25A1, in proliferating cells has been suggested as a mitochondrial specific approach to tumor treatment [89, 90].

## 4. Conclusion

Mitochondria have a critical role to play in the successful conquest of cancer. Further and deeper investigations of this organelle assure profound insights into the missing molecular mechanisms of malignancy. The often referred to “power-house of the cell” is beginning to look more like a well ordered neighborhood of sprawling metabolic mansions. Some areas contain décor dating from the earliest of antiquities, while others have yet to be opened and thoroughly explored for the elusive, but ultimate answers to cancer biology; however, many have hurried through the biological lobby of this complex like tourists on a bus schedule. We must now committee to taking the grand tour; more magnificent biological vistas await. Mitochondria may yet be found to be the master of the cellular orchestra.
